# Effect of ultrasonic and Nd: Yag laser activation on irrigants on the push-out bond strength of fiber post to the root canal

**DOI:** 10.1590/1678-7757-2018-0420

**Published:** 2019-05-30

**Authors:** Funda Fundaoğlu Küçükekenci, Ahmet Serkan Küçükekenci

**Affiliations:** 1 Ordu Public Oral Health Hospital, Ordu, Turkey.; 2 Ordu University, Faculty of Dentistry, Department of Prosthodontics, Ordu, Turkey.

**Keywords:** Irrigation, Lasers, Dental cement, Bonding

## Abstract

**Objective:**

This *in vitro* study aimed to compare the efficacy of irrigants using various irrigation activation methods to the push-out bond strengths of fiber post to root canal luted with self-adhesive resin cement (SARC).

**Methodology:**

Forty-eight decoronated human canines were used. The specimens were divided into four groups corresponding with the post-space irrigation process and were treated as follows: distilled water (DW) (Control) group received 15 mL of DW; sodium hypochlorite (NaOCl)+ethylenediaminetetraacetic acid (EDTA) group was treated with 5 mL of 5.25% NaOCl, 5 mL of 17% EDTA, and 5 mL of DW; passive ultrasonic irrigation (PUI) group was treated with 5 mL of 5.25% NaOCl, 5 mL of 17% EDTA, and 5 mL of DW, and each irrigant was agitated with an ultrasonic file; and laser activated irrigation (LAI) group was treated with 5 mL of 5.25% NaOCl, 5 mL of 17% EDTA, and 5 mL of DW, and each irrigant was irradiated with Nd: YAG laser. Fiber posts were luted with SARC, and a push-out test was performed. Data was analyzed using one-way analysis of variance and Tukey HSD test.

**Results:**

The bond strength values for the groups obtained were as follows: Control (10.04 MPa), NaOCl+EDTA (11.07 MPa), PUI (11.85 MPa), and LAI (11.63 MPa). No statistically significant differences were found among all experimental groups (p>0.05). The coronal (12.66 MPa) and middle (11.63 MPa) root regions indicated a significantly higher bond strength compared with the apical (9.16 MPa) region (p<0.05).

**Conclusions:**

Irrigant activation methods did not increase the bond strength of fiber post to canal.

## Introductıon

Endodontically treated teeth with excessive loss of coronal structure can be restored by using fiber posts. Some advantages of fiber posts include credible mechanical properties and lower modulus of elasticity, similar to that of dentin, and thus there exists little risk of causing root fracture. Another advantage is that it is post-translucent, which allows the passage of light which is necessary for cementing polymerization, allowing the post to be connected to the dentin wall.^1^

When the post is luted to the root canal, two interfaces occur. One of them is between the post and the cement, and the other is between the cement and the root dentin.^2^

Most commonly reported mode of clinical failure in fiber post is debonding from root dentin.^3^ The main problem that is believed to adversely affect the bonding strength of fiber post is the blockage of cement adhesion by obstruction of the dentinal tubules by the Gutta-percha and sealer remnants, dentine debris and smear layer.^4^ In addition to external factors such as irrigants, types of adhesives, and endodontic sealers, dentin-related factors such as dentin status and orientation of dentin tubules also affect these interfaces.^5^

Irrigation after post space preparation procedures help remove the smear layer and may increase the strength between the cement bond and the root canal dentin^6^. Frequently suggested irrigation protocols are 5.25% sodium hypochlorite (NaOCl) and 17% ethylenediaminetetraacetic acid (EDTA). NaOCl is used to remove the organic content of the smear layer while EDTA is often used to remove the inorganic content of the smear layer.^7^ Unfortunately, irrigants are unable to completely remove the filling material from the canals.^8^ Therefore, several activation techniques were developed to more effectively remove pulp tissue and microorganisms, smear layer, and dentin debris from the root canal system, such as passive ultrasonic irrigation (PUI) and laser activated irrigation (LAI).^9^

PUI is the ultrasonic activation of an irrigant in the root canal via an ultrasonically oscillating small file placed in the root canal after the root canal has been shaped.^10^ Recently, LAI has been introduced as an activation method of irrigation solutions that uses transfer of pulsed energy by means of various laser systems.^11^

Self-adhesive resin cement (SARC) systems are utilized to overcome the technical problems of multi-step applications and to shorten the duration of clinical application. The main adhesive characteristic of SARC is attributable to a chemical reaction between phosphate methacrylates and hydroxyapatite; this cement presents limited infiltration into the tooth tissue.^12^

The connection between post-dentin and cement is important for restoration stability and longevity.^13^ Hence the effect of different irrigation methods on post-dentin bonding strength should be investigated after post-space preparation. Therefore, in this study, we aimed to evaluate the effect of irrigant activation techniques on the push-out bond strength of fiber posts. The null hypothesis tested was that irrigant activation techniques do not affect the push-out bond strengths of fiber posts to root dentin.

## Methodology

### Specimen preparation

Forty-eight freshly extracted human maxillary canines were selected for this study. Teeth with a single straight root canal and developed apices fulfilled the inclusion criteria. All teeth were stored in 0.1% thymol until the experimental procedure. Periapical radiographs were taken from both the mesiodistal and buccolingual sides to ensure that there was only one straight canal in each tooth. Teeth that presented prior endodontic treatment and fracture lines were excluded from this study.

Each specimen was decoronated using a low-speed saw (Mecatome T180; Presi, Eybens, France) under water cooling to provide a uniform root length of 15 mm. All root canals were prepared to size R50 with the RECIPROC system (VDW, Munich, Germany). Irrigation was made using 5 mL of 5.25% NaOCl with 5 mL of 17% EDTA solution for 1 min and 5 mL of 5.25% NaOCl between instrument changes. Distilled water (DW; 5 mL) was used for a final irrigation. Finally, the canals were dried using sterile paper points. All instrumented teeth were obturated with gutta-percha cone and AH Plus sealer (Dentsply De Trey, Konstanz, Germany) with use of the cold lateral compaction technique, and the canals were covered with temporary filling material (Cavit-G; 3M ESPE, Seefeld, Germany). All specimens were stored at 37°C and 100% humidity for 7 days, after which the temporary fill was removed. The filling material in all canals was removed using a standard technique. Reinstrumentation of the canal was initiated by removing the filling material on the 3-mm cervical portion with Gates Glidden burs size #4 (Dentsply-Maillefer, Ballaigues, Switzerland). In total, 12 mm of filling material was removed. Lastly, the final shape of the root canals was prepared with a blue post drill (Reforpost; Angelus, Londrina, PR, Brazil).

The teeth were divided into four groups (*n*=12) according to the irrigation process:

Control group: 15 mL of DW was used for irrigation of post spaces.

NaOCl + EDTA group: 5 mL of 5.25% NaOCl, then 5 mL of 17% EDTA and 5 mL of DW were used to irrigate the post spaces.

PUI group: 5 mL of 5.25% NaOCl, then 5 mL of 17% EDTA and 5 mL of DW was used for irrigation of post spaces. Each irrigant was agitated with an ultrasonic system handpiece (VDW Ultra; VDW, Munich, Germany) equipped with a size 25 IRRI S smooth wire (VDW; Endo Ultrasonic Files, Endodontic Synergy, Munich, Germany) at 1 mm short of the working length, as previously described. The irrigation was ultrasonically applied to the root canals for 3 min in total along with 5 mL of 17% EDTA, 5 mL of 5% NaOCl, and 5 mL DW for 1 min (3 cycles of 20 s) for each irrigant according to previous studies.^14^ ^-^ ^19^

LAI group: 5 mL of 5.25% NaOCl, 5 mL of 17% EDTA and 5 mL of DW was used for irrigation of post spaces. Each irrigant was fully activated for 1 min (3 cycles of 20 s) with 1064-nm wavelength Neodymium-doped Yttrium Aluminum Garnet (Nd: YAG) laser (Dekalaser SmartFile; DEKA, Calenzano, Florence, Italy) at 1 W/cm^2^ Power (20 Hz Frequency, 50 mj/cm^2^ Energy density) with a pulse duration of 50 μs by a non-cooled handpiece with 300 μm optical fiber.

After the post-space irrigation procedure, root canals were dried using paper point. The post spaces were covered with SARC (RelyX U200; 3M ESPE, St. Paul, MN, USA) using Automix tips (3M ESPE, St. Paul, MN, USA) in accordance with the manufacturer's instructions. Fiber posts with a diameter of 1.5 mm (Reforpost; Angelus, Londrina, PR, Brazil) were placed with mild pressure, and the SARC was polymerized with a light-emitting diode unit (Elipar S10, 3M ESPE, Neuss, Germany). All specimens were stored at 37°C and 100% humidity for 24 hours.

The specimens were buried in acrylic blocks to be cut with a low-speed saw. The samples were horizontally cut to obtain 1-mm sections. Six samples from each root were obtained. The samples were then separated into two pieces belonging to the coronal, middle and apical parts of the root. The 2^nd^, 4^th^, and 6^th^ slices were selected for a push-out test of the samples obtained from the coronal, middle and apical parts.

### Push-out test

The push-out bond strength was measured using a universal testing machine (Autograph AGS X; Shimadzu Co, Japan). The push-out test was applied at 0.5 mm/min using a 1-mm diameter metallic plunger from the apical to the coronal direction until the post was dislodged. Peak force, namely the force applied at the point of extrusion of the post segment from the test specimen, was taken as the point of bond failure and was recorded and measured in Newtons (N). The maximum failure load was converted to megapascals (MPa) for each slice and was adjusted for the total bonding area (mm^2^) of each segment. The post diameter was measured with a digital caliper at both coronal and apical posts as well as the dentin surface sections. The total bonding area was calculated as (p [R+r] [h^2^+(R−r)^2^]^0.5^), where R = post radius, r = apical post radius, and h = slice thickness.

### Statistical analysis

**Figure 1:**
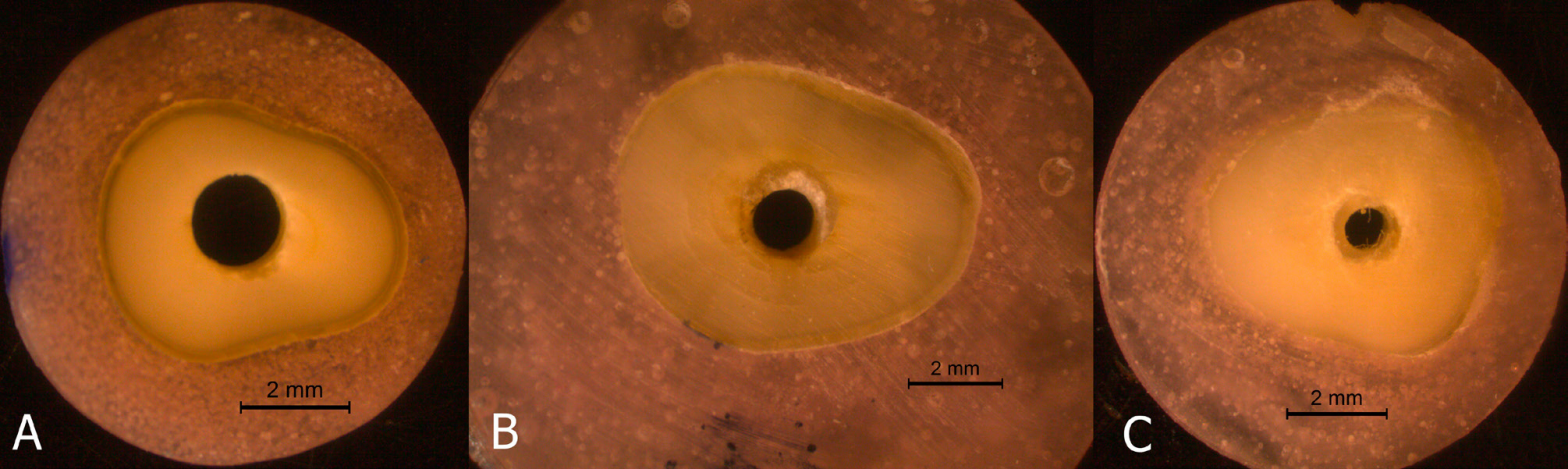
Stereomicroscope image of failure modes. (A) Adhesive failure between cement and dentin. (B) Mixed failure. (C) Cohesive failure. (x25)

The push-out bond strength (MPa) data were analyzed by one-way analysis of variance (ANOVA). Finally, the mean SBS values were compared using Tukey multiple comparison tests (*p*=0.05). The complete computational work was performed with the aid of statistical software (IBM Corp. Released 2011. IBM SPSS Statistics for Windows, Version 20.0. Armonk, NY, USA). Debonded surfaces were accessed using stereomicroscope (Leica SP1600; Leica, Wetzlar, Germany) at x25 magnification. Failure modes of the specimens were classified as follows: 1. Adhesive (failure at interface between adhesive resin and dentin); 2. Mixed (exhibits some cohesive failure and some adhesive failure); 3. Cohesive (cohesive failure within resin) (Figure 1).

## Results

The means and standard deviations of the push-out bond strength values are presented in Table 1. The bond strength values (MPa) for the groups were as follows: Control (10.04±4.48), NaOCl+EDTA (11.07±2.72), PUI (11.85±5.45) and LAI (11.63±4.40). No statistically significant differences were found between groups (*p*>0.05).

**Table 1:** Mean±SD of push-out bond strength values in different groups and three regions of root canal

	N	Control	NaOCl + EDTA	PUI	LAI	Total
Coronal	12	11.31^a,A^ (4.31)	12.27^a,B^ (2.57)	13.91^a,B^ (7.34)	13.14^a,A^ (3.3)	12.66^B^ (4.69)
Middle	12	9.84^a,A^ (3.98)	11.42^a,AB^ (1.91)	12.98^a,AB^ (2.73)	12.28^a,A^ (4.16)	11.63^B^ (3.43)
Apical	12	8.98^a,A^ (5.14)	9.53^a,A^ (2.99)	8.66^a,A^ (4.02)	9.47^a,A^ (5.04)	9.16^A^ (4.26)
Total	36	10.04^a^ (4.48)	11.07^a^ (2.72)	11.85^a^ (5.45)	11.63^a^ (4.40)	

* Results of Tukey post-hoc comparisons were shown as superscripts and values having same letters are not significantly different (p>0.05)

In accordance with the root region, the means and standard deviations of the push-out bond strength values are presented in Table 1. The bond strength values (MPa) for the root regions were as follows: apical (9.16±4.26), middle (11.63±3.43), and coronal (12.66±4.69). The apical root region showed a significantly lower bond strength compared to the coronal and middle root regions (p<0.05). No statistically significant differences were found between the other two regions (p>0.05).

Intragroup comparisons revealed that bond strength values decreased in the coronal-to-apical direction, there was no significant difference in Control and LAI groups (*p*>0.05) but apical root regions were significantly lower than the coronal root region in NaOCl+EDTA and PUI groups (*p*<0.05) (Table 1).

The frequency of each type of bond failure mode is given in Table 2. The most common failure mode was adhesive (51.4%), followed by mix failure between dentin and resin cement (41.7%) and cohesive failure in the resin cement (6.9%).

**Table 2:** Mode failure percentages with respect to post space irrigation procedure

Mode of failure	Control	NaOCl + EDTA	PUI	LAI	Total
Adhesive	15 (41.7%)	19 (52.7%)	18 (50%)	22 (61.1%)	74 (51.4%)
Mixed	18 (50%)	15 (41.7%)	15 (41.7%)	12 (33.3%)	60 (41.7%)
Cohesive	3 (8.3%)	2 (5.6%)	3 (8.3%)	2 (5.6%)	10 (6.9%)
Total	36	36	36	36	144

## Dıscussıon

In many cases, treatment failure caused by cementation failure could be restored with a post after endodontic treatment.^1^ The purpose of this *in vitro* study was to evaluate the effects of various irrigation methods on the push-out bond strengths of fiber posts to root dentin with SARC. Our results revealed that the examined irrigation methods slightly affected fiber post bond strength, but this was not found to be statistically significant. In accordance with the results, the null hypothesis was accepted.

After the post cavity was prepared, the presence of a smear layer consisting of sealer, gutta-percha, and debris was observed on the dentin surfaces examined with a scanning electron microscope.^20^ When etch-and-rinse systems are used, removing the smear layer becomes essential in order to achieve a hybrid layer.^4^ When endodontic treatment is applied for the first time or during retreatment, a combination of NaOCl and EDTA is considered as effective irrigation for smear removal but smear layer cannot be completely removed.^21^ Therefore, self-adhesive cement does not require hybrid layer formation and is considered as an advantage for post retention.^12^ Therefore, the effects of the irrigation method can cause controversial results on the bond strength when SARC is used. Ertaş et al.^22^ (2014) demonstrated that irrigation with the combination of NaOCl and EDTA negatively affects the bond strength between the post and root dentin. These results may explain why the removal of the smear layer with NaOCl and EDTA may cause extreme etching and reduced bond strength.^23^ Some researchers reported that irrigation with NaOCl and EDTA combinations did not have an adverse effect on post-dentin bonding strength in their study, while other researchers reported an increase in bonding strength when post-space irrigation was performed with NaOCl/EDTA combination.^24^ The results of our study showed that the NaOCl/EDTA combination did not affect bond strength of the fiber posts to the root canals.

The use of PUI and LAI for disinfection has become increasingly widespread in recent years due to their effect on irrigants.^14^ The energy produced by the lasers is vertically and horizontally transferred to the irrigant via the fiber cable. This energy causes the formation of bubbles in the irrigant, which causes cavitation through pressure on the root dentin.^25^ Nd: YAG laser can be used to remove the smear layer from the hard tissues due to its low wavelength. Additionally, the heat generated by the laser accelerates the decomposition of NaOCl into chlorine and oxygen ions, thereby increasing the effect of NaOCl.^26^ Ultrasonic activation causes an acoustic flow in the irrigation solution due to rapid circular movement.^14^ Although the effectiveness of LAI and PUI for irrigation activation in eliminating bacteria is well known, the effect on the bond strength of post to root dentin after use is not known.^27^ The findings of this study showed that the combination of NaOCl/EDTA activation with Nd: YAG laser and PUI had no significant effect on bonding strength when compared with the NaOCl/EDTA combination alone.

Some investigations recommend PUI for 20 seconds.^28^ ^–^ ^30^ However, some studies^19^ ^–^ ^24^ recommend complete PUI 60 seconds, especifically 3 cycles of 20 s. In this study, we preferred the 3 cycles of 20 s (totally 60 s) based on these studies.

A few studies have evaluated the effect of different irrigation methods on the bond strength of post to dentin with luting SARC.^31^ ^,^ ^32^ Bitter et al.^32^ (2013) indicated that the use of combined NaOCl/EDTA solutions positively affected SARC bond strength. However, in accordance with our results, the combination of NaOCl and EDTA has not been observed to increase SARC bonding strength, which can be explained by the assumption that when activation methods are combined with the irrigant, the SARC may adversely affect bonding strength. Some researchers have found that removal of the smear layer negatively affects the adhesion of cement when using self-adhesive systems.^33^

Different bonding strengths can be seen in the different parts of the root during treatment procedures that use SARC and are expected to decrease the bonding strength from the coronal region to the apical region.^34^ In our study, as in previous reports,^6^ ^,^ ^24^ the push-out strength of fiber post was affected significantly by the root region. Push-out bond strength values in the coronal and middle regions were significantly better than in the apical region. This phenomenon is caused by the distribution and density of dentinal tubules in the different regions of the root. There are reports that tubule density in the coronal region is greater than in the apical region and that the tubule diameter decreases in the apical direction. The other clinical factors such as negative C factors and limited access to the apical region also lead to reduced bond strength at the apical region^34^. Additionally, there were no significant differences in the same region of all groups in our study.

Generally, alternative irrigation activation techniques have been proposed to improve the efficacy of irrigants within the root canal system.^35^ A limitation of the current study was the assessment of only two activation techniques. Further investigation is required to determine the effects of different irrigation activation techniques to obtain a better bond strength of fiber post to root canal.

According to the results of our study, when fiber post luted to root dentin with SARC, none of the irrigation methods we examined have the advantage over the other.

## Conclusions

The findings of this study showed that irrigant activation methods did not increase the bond strength of fiber post to dentin luted with SARC, and apical root regions exhibited a significantly lower bond strength than the coronal and middle root regions.
